# Autonomic Dysfunction After Guillain–Barré Syndrome: Evidence from Comprehensive Autonomic Testing

**DOI:** 10.3390/jcm15155963

**Published:** 2026-07-30

**Authors:** Rafaella Theologou, Theodora Barkoula, Christiana Ioannou, Antonis Pilavas, Sakis Lambrianides, Nikolaos Grigoriadis, Georgios M. Hadjigeorgiou, Panagiotis Zis

**Affiliations:** 1 2nd Department of Neurology, Aristotle University of Thessaloniki, 54124 Thessaloniki, Greece; 2 Department of Neurology, Nicosia General Hospital, 2031 Nicosia, Cyprus; 3 First Department of Anaesthesiology, Aretaieion Hospital, Medical School, National Kapodistrian University of Athens, 11527 Athens, Greece; 4 Medical School, University of Cyprus, Shacolas Educational Centre for Clinical Medicine, 2029 Nicosia, Cyprus; 5 Apollonion Private Hospital, 2054 Nicosia, Cyprus

**Keywords:** Guillain–Barre syndrome, dysautonomia, autonomic dysfunction, autonomic testing

## Abstract

**Background/Objectives:** Guillain–Barré syndrome (GBS) is an acute immune-mediated disorder of the peripheral nervous system. Autonomic involvement in GBS occurs during the acute phase and may persist into the recovery phase. This study aimed to investigate the autonomic nervous system’s function in GBS sufferers. **Methods:** A cross-sectional exploratory study was conducted at the Clinical Neurophysiology Laboratory, Medical School, University of Cyprus. Eligible patients were previously hospitalized with GBS. Autonomic nervous system assessment included the Ewing battery using the Finapres Nova system as well as sudomotor assessment. **Results:** Seventeen patients were recruited (53% female; mean age 58.7 ± 14.1 years). The mean follow-up time since diagnosis was 15.5 ± 13.2 months (range 1–45 months). At follow-up, 4/17 patients (24%) demonstrated an abnormal heart rate (HR) response to standing, 3/17 patients (18%) exhibited an abnormal systolic blood pressure (BP) response to standing, and 4/17 patients (24%) exhibited an abnormal diastolic BP response to standing. One patient (6%) demonstrated an abnormal HR response during deep breathing, while another showed an abnormal diastolic BP response during the handgrip test. Overall, 6/17 patients (35%) showed abnormal findings in at least one component of the Ewing battery, and 3/17 (18%) fulfilled the criteria for definite generalised dysautonomia. Two patients (12%) demonstrated abnormal sympathetic skin response (SSR) results; both fulfilling the criteria for dysautonomia based on the Ewing battery. **Conclusions:** Autonomic dysfunction may be present in GBS patients beyond the acute phase, even in the absence of overt autonomic symptoms. Larger prospective studies with serial autonomic assessments are required to better characterise the long-term course and clinical significance of dysautonomia in GBS.

## 1. Introduction

Guillain–Barré syndrome (GBS) is an acute immune-mediated disorder of the peripheral nervous system and is the leading cause of acute non-traumatic neuromuscular paralysis worldwide [1]. The annual incidence of GBS is approximately 1–2 cases per 100,000 individuals, with higher rates among older adults and males [2]. Clinically, GBS presents with rapidly progressive weakness, sensory disturbances, and diminished or absent reflexes, typically following a respiratory or gastrointestinal infection [1,3]. Weakness most often begins in the lower limbs and ascends, potentially involving the arms, facial muscles, or swallowing function. Reflexes are generally reduced or absent, although this may not be apparent in the early stages. Sensory disturbances are common but tend to be less pronounced than motor weakness [1,3]. As the condition progresses, cranial nerve involvement may occur, leading to facial weakness or difficulties with speech and swallowing.

GBS presents in several clinical forms, reflecting differences in the structures targeted by the immune response. The demyelinating form, acute inflammatory demyelinating polyneuropathy (AIDP), is most commonly observed in Europe and North America and primarily affects the myelin sheath of peripheral nerves [1]. Myelin damage disrupts the efficient transmission of electrical signals, resulting in slowed conduction. In contrast, axonal variants such as acute motor axonal neuropathy (AMAN) and acute motor and sensory axonal neuropathy (AMSAN) involve direct injury to nerve fibers. These forms are more frequently reported in Asia and Latin America and are often associated with antibodies directed against gangliosides such as GM1 and GD1a [1,3]. Clinically, axonal variants tend to follow a more severe course and may be associated with delayed recovery. Another well-recognized variant is Miller Fisher syndrome (MFS), characterized by the triad of ophthalmoplegia, ataxia, and areflexia, and strongly associated with anti-GQ1b antibodies [3]. Additional regional variants include the pharyngeal–cervical–brachial form and paraparetic presentations, further illustrating the clinical diversity of the syndrome [1].

Although motor symptoms are well established, involvement of the autonomic nervous system (ANS) is also frequent and clinically significant. Autonomic dysfunction occurs in approximately 50–66% of patients, with some studies reporting involvement in up to two-thirds of individuals with GBS [4,5,6]. Dysautonomia may affect cardiovascular, gastrointestinal, sudomotor, and genitourinary systems, resulting in blood pressure instability, arrhythmias, tachycardia, gastroparesis, constipation or diarrhoea, and urinary disturbances [4,5]. Cardiovascular autonomic dysfunction is particularly critical, as severe fluctuations in heart rate and blood pressure can be life-threatening and contribute significantly to morbidity and mortality [5]. Respiratory muscle weakness is another major complication, with 20–30% of patients requiring mechanical ventilation during the acute phase [2,6]. The presence of autonomic dysfunction is associated with a more severe disease course, and up to 75% of patients with dysautonomia may require ventilatory support and intensive care management [5]. Autonomic involvement is also linked to prolonged recovery and increased healthcare burden [5,7,8].

Given the essential role of the ANS in maintaining physiological homeostasis, elucidating the mechanisms and clinical implications of autonomic dysfunction in GBS is crucial for early recognition, close monitoring, and effective management. The aim of this study was to characterize the presence and pattern of autonomic abnormalities in patients following GBS.

## 2. Materials and Methods

This was a cross-sectional exploratory study. Patients hospitalized with GBS from 2020 to 2026 being under regular follow-up underwent a full autonomic assessment. The study was conducted at the Clinical Neurophysiology Laboratory of the Medical School of the University of Cyprus at Nicosia General Hospital.

Inclusion criteria comprised age 18 years or older with a diagnosis of GBS by the treating neurologist based on accepted clinical criteria [2]. Patients with neurological or psychiatric disorders associated with severe cognitive impairment, rendering them unable to fully cooperate with the diagnostic procedures, were excluded. Demographic features, clinical characteristics, laboratory findings, and GBS Disability Scale (GBS-DS) [9] scores at onset, discharge and follow-up were collected for all patients.

All participants underwent bilateral sensory nerve conduction studies of the superficial radial, ulnar, median, and sural nerves, as well as motor nerve conduction studies of the median, ulnar, and tibial nerves. The examination was performed in a quiet, comfortable environment using a Natus EDX electromyography (Natus, Middleton, WI, USA) with surface electrodes. In all measurements, the temperature was above 32 °C for the upper limbs and above 31 °C for the lower limbs. For each nerve, distal latency, amplitude, and conduction velocity were recorded. Results were interpreted using in-house laboratory reference values for the corresponding nerves based on 210 healthy controls stratified per age. Based on the clinical and electrophysiological findings, patients were classified into subtypes including acute motor axonal neuropathy (AMAN), acute motor and sensory axonal neuropathy (AMSAN), acute inflammatory demyelinating polyneuropathy (AIDP), or MFS.

The sural-to-radial amplitude ratio (SRAR) was also included in the protocol as an electrophysiological parameter to assess primary or secondary axonal damage. Values below 0.21 were considered abnormal and supportive of the diagnosis [10,11].

Autonomic nervous system (ANS) function was evaluated using Sympathetic Skin Response (SSR), SUDOSCAN, and cardiovascular autonomic reflex tests (Ewing battery) performed with the Finapres system.

Recording of the SSR was obtained from the palm of the hand and the sole of the foot to the opposite site of the stimulated. A single electrical stimulus of 50–100 mA was given. The procedure was then repeated 3 times with at least 10 s intervals between the stimulations [12,13,14]. Results were interpreted using in-house laboratory reference values for the corresponding nerves based on 177 healthy controls. Absent responses or amplitudes <160μV in the hand or <125μV in the foot, where characterised as abnormal.

Sudomotor function was tested using the SUDOSCAN device (Impeto Medical, San Diego, CA, USA) to evaluate sweat gland activity, which indirectly reflects autonomic and small fiber nerve function. The examination involved placing participants’ palms and soles on stainless steel electrodes, where a low direct current was applied to measure electrochemical skin conductance (ESC), expressed in microsiemens (µS), reflecting chloride ion release from sweat glands [15,16]. Diagnosis of sudomotor dysfunction is based on ESC thresholds established in previous studies. Values greater than 70 µS for the feet and 60 µS for the hands are considered indicative of normal sudomotor function. Intermediate values (50–70 µS for the feet and 40–60 µS for the hands) suggest moderate dysfunction, while values below 50 µS for the feet and 40 µS for the hands are consistent with severe impairment of sweat gland function [15].

The Ewing battery is a standardised set of five simple, non-invasive cardiovascular reflex tests used to evaluate autonomic nervous system (ANS) function, particularly in diagnosing dysautonomia [17,18]. These tests are performed using continuous cardiovascular monitoring with Finapres (Finapres-NOVA, Enschede, The Netherlands) system, a non-invasive beat-to-beat blood pressure monitor that uses a finger cuff and the volume-clamp method (Jan Peñáz principle) to track arterial pressure continuously. It works by detecting changes in finger arterial volume via photoplethysmography and dynamically adjusting cuff pressure to keep arterial volume constant, allowing real-time measurement of blood pressure and heart rate variability during each manoeuver. All measurements were conducted according to the manufacturer’s instructions.

The Valsalva manoeuvre was performed by asking the subject to exhale against a pressure of 40 mmHg for 15 s through a mouthpiece, and the Valsalva ratio was calculated as the ratio of the longest R–R interval after the manoeuvre to the shortest R–R interval during the manoeuvre. Deep breathing testing was conducted at a controlled respiratory rate of six breaths per minute (5 s inspiration and 5 s expiration), and heart rate variability was assessed using the expiratory-to-inspiratory (E/I) ratio. The sustained isometric handgrip test was performed by maintaining handgrip at 30% of maximal voluntary contraction using a dynamometer for up to 5 min, while the increase in diastolic blood pressure was recorded. Heart rate response to standing was evaluated using the 30:15 ratio, defined as the ratio of the longest R–R interval around the 30th beat to the shortest R–R interval around the 15th beat after standing from the supine position. Finally, blood pressure response to standing was assessed by measuring systolic blood pressure in the supine position and again after standing, with the decrease in systolic blood pressure representing the orthostatic response. The proposed thresholds for each test were adopted from the systematic review by Barkoula et al. [17]. Each test is categorised as normal, borderline, or abnormal according to established thresholds. A finding of one abnormal test is generally considered indicative of early autonomic involvement, whereas the presence of two or more abnormal tests is consistent with definite autonomic dysfunction.

For the Ewing battery the patients should avoid nicotine, caffeine, and large meals for at least 2 h prior to testing. Strenuous exercise should be avoided for at least 24 h. Record all medications, and if feasible, withhold autonomically active medications for at least five half-lives prior to assessment. This includes medications such as beta-blockers [17].

Statistical analysis was performed using Statistical Package for the Social Sciences (SPSS, version 29.0). The analysis was mainly descriptive. Comparisons between patients with definite autonomic dysfunction and patients without (as determined by the Ewing battery) were conducted using the chi-square test or Fisher’s exact test for categorical variables, and the independent t-test or Mann–Whitney U test for continuous variables, as appropriate. A *p* value < 0.05 was considered statistically significant.

## 3. Results

### 3.1. Demographics and Clinical Characteristics

Twenty-six consecutive GBS patients were identified from our records. Of these, one patient had died, two patients had moved to a different country, one patient failed to attend two scheduled autonomic assessment appointments, one was lost to follow-up, one was still undergoing rehabilitation, and three declined to participate because they felt they were doing well. In total, 17 patients (response rate 65%) participated in this study (53% females, mean age 58.7 ± 14.1 years, range 36 to 77 years). The mean follow-up time since diagnosis, when the autonomic testing took place, was 15.6 ± 13.1 months (range 2–45 months). Comorbidities of the patients were unremarkable, apart from only one patient who also had diabetes mellitus.

Twelve patients (71%) were diagnosed with typical GBS [8 patients (47%) with AIDP, 3 patients (18%) with AMAN, whereas for 1 patient no baseline NCS was available for classification], 2 patients (12%) with MFS, and 3 patients (18%) with GBS/MFS. Demyelinating features were present in half of the patients. All patients with MFS or the MFS/GBS variant were females.

Thirteen out of 17 patients (76%) had an antecedent infection, of which 53% (9 patients) had a respiratory infection and 18% (3 patients) had a gastrointestinal infection. The mean time from antecedent infection to disease onset was 14.8 ± 10.1 days.

Fourteen out of 17 patients (82%) had albuminocytologic dissociation, with a mean cerebrospinal fluid cell count of 2.7 ± 2.3 (range 0 to 8) and mean cerebrospinal fluid protein of 116 ± 77 mg/dL. Twelve patients (70%) received intravenous immunoglobulin (IVIG) alone as first-line treatment, four patients (24%) received both IVIG and plasma exchange (PLEX), and one patient (6%) received PLEX alone.

Two patients (12%) required admission to the ICU. One was categorized as AMAN and the other was unspecified. The mean GBS-DS score was 3.4 ± 0.8 at admission, 3.0 ± 1.0 at discharge, and 1.1 ± 1.2 at follow-up. Twelve patients (71%) have fully recovered or have GBS-DS score 1.

At follow-up, no patients complained of neuropathic pain. The nerve conduction studies showed three patients (18%) with residual large fiber involvement with abnormal SRAR.

### 3.2. Ewing Battery

At follow-up, 4 (24%) patients showed an abnormal HR response to standing, 3 (18%) patients showed an abnormal SBP response to standing, 4 (24%) patients showed an abnormal DBP response to standing, 1 (6%) patient showed an abnormal HR response during deep breathing and two (12%) abnormal DBP response during handgrip. Of the 14 patients who were able to perform the Valsalva manoeuvre, all showed normal responses. In total, 7 (41%) patients showed an abnormal response in at least one test of the Ewing battery, and 3 patients (18%) fulfilled the criteria for dysautonomia (among which the one patient who also had diabetes).

### 3.3. Sudomotor Assessment

Two patients (12%) had abnormal SSRs, both fulfilling the criteria for dysautonomia based on the Ewing battery.

The mean ESC for the feet was 68.7 ± 8.9 µS (range 50–81) and for the hands was 77.6 ± 9.1 µS (range 57–87). Ten patients (59%) showed moderate sudomotor dysfunction based on the ESC of the feet and two patients (12%) showed moderate sudomotor dysfunction based on the ESC of the hands. No patients showed severe sudomotor dysfunction in either the hands or the feet based on the ESC. All three patients that were classified as suffering from dysautonomia based on the Ewing battery showed abnormal ESC of the feet.

Table 1 summarizes the clinical and electrophysiological characteristics of the cohort, whereas Table 2 presents the detailed results for each patient.

### 3.4. Determinants of Dysautonomia

We compared patients with definite autonomic dysfunction at follow-up, as determined by the Ewing battery (*n* = 3), with those without autonomic dysfunction (*n* = 14). Patients with dysautonomia were assessed earlier after GBS diagnosis (8.0 ± 2.0 vs. 17.2 ± 14.0 months; *p* = 0.375) and were older (71.3 ± 3.2 vs. 55.9 ± 3.7 years; *p* = 0.068). They also had higher GBS-DS scores at admission (4.0 ± 0.0 vs. 3.3 ± 0.8), discharge (3.7 ± 0.6 vs. 2.9 ± 1.0), and follow-up (2.3 ± 2.1 vs. 0.8 ± 0.8), as well as lower SRAR values (0.21 ± 0.32 vs. 0.49 ± 0.22). However, none of these differences reached statistical significance, likely because of the small sample size. Sex, ICU admission, and GBS subtype were also not associated with the presence of dysautonomia.

## 4. Discussion

Studies assessing autonomic dysfunction in patients with GBS are limited. Most available studies have focused on autonomic dysfunction during the acute phase of the disease, with some reporting autonomic involvement in up to 70% of patients [5,6,7]. Singh et al. reported transient and generally mild autonomic dysfunction involving both the sympathetic and parasympathetic nervous systems, predominantly occurring during the peak phase of paralysis [4].

Only a few studies have evaluated autonomic nervous system function following a substantial interval after the acute phase of GBS using objective measurements. Flachenecker et al., in a longitudinal study of 13 patients with GBS, reported that none of the patients had dysautonomic symptoms after one year of follow-up [19]. Koeppen et al. assessed patients between 7 and 86 months after disease onset using power spectrum analysis (PSA) with the ProSciCard system to evaluate heart rate variability (HRV), including the 30:15 ratio in response to standing and the E/I ratio during deep breathing. Systolic and diastolic blood pressure were evaluated using the Finapres device. The authors demonstrated pathological blood pressure responses to standing in 27 out of 33 patients [20]. In contrast, HRV measures were largely preserved: the 30:15 ratio was within the normal range in all but one patient, and reduced resting HRV was observed in only one patient. Most patients demonstrated elevated coefficient of variation (CoV) and root mean square of successive differences (RMSSD) values at rest and during deep breathing, suggesting preserved or enhanced cardiovagal modulation.

In our study, blood pressure abnormalities were also observed in four patients: three patients (18%) showed an abnormal systolic blood pressure response to standing, while four patients (24%) demonstrated an abnormal diastolic blood pressure response to standing.

Zaeem et al. [7] performed autonomic nervous system assessment in 26 patients 3–8 years after the onset of GBS. Abnormal heart rate variability during deep breathing was identified in one patient (4%), while an abnormal Valsalva ratio was found in four patients (15%). Sympathetic abnormalities were more common, with orthostatic hypotension detected in 15 patients (58%) and an abnormal blood pressure response during sustained isometric handgrip testing observed in 17 patients (65%) [7].

In our study, abnormal heart rate variability during deep breathing was observed in one patient (6%), a finding comparable to that reported by Zaeem et al. [6], whereas all patients who were able to perform the Valsalva manoeuvre had normal responses. Orthostatic hypotension was identified in 4 patients (24%), substantially fewer than the 58% reported by Zaeem et al. [6]. Similarly, an abnormal blood pressure response during handgrip testing was observed in only one patient (6%), compared with 65% in their cohort.

A novelty of our study is that we conducted the full Ewing battery and managed to describe how many patients fulfilled the gold standard criteria for definite autonomic dysfunction. In total, 18% of patients fulfilled these criteria. Moreover, in our department we use a more objective and comprehensive approach, which apart from the Ewing battery includes sudomotor assessment through SUDOSCAN and SSR. This allowed the detection of subtle or subclinical abnormalities that might otherwise remain unrecognized. In fact, 12% of patients demonstrated abnormal sudomotor findings, which were concordant with abnormalities detected by other autonomic tests.

Our results should be interpreted with caution given the limitations of our study design. First, the total sample size was relatively small, reflecting the rarity of GBS and the challenges associated with long-term follow-up in this patient population. Although patients with dysautonomia in our cohort tended to be older and had higher disability scores throughout the disease course, these associations did not reach statistical significance, most likely due to the limited sample size. Nevertheless, despite the small cohort, comprehensive multimodal autonomic assessment allowed the identification of autonomic abnormalities in a substantial proportion of patients, supporting the clinical relevance of our findings. Second, as participation required attendance at a follow-up autonomic assessment, patients who had died, relocated, remained in rehabilitation, were lost to follow-up, or declined participation were not included, introducing the potential for recruitment bias and limiting the generalizability of the findings. Third, the cross-sectional nature of the study limits conclusions regarding the temporal evolution of autonomic dysfunction after the acute phase of GBS. Patients were assessed at different time points following diagnosis, introducing heterogeneity in recovery stages and potentially influencing autonomic test results. However, the aim of the present study was not to investigate the temporal evolution of autonomic dysfunction from the acute phase to recovery, but rather to characterize the presence and pattern of autonomic abnormalities in patients following GBS and to provide a pragmatic overview of autonomic involvement across a broad spectrum of post-GBS recovery periods encountered in real-world clinical practice. Finally, baseline autonomic assessments during the acute phase were not available for comparison, limiting our ability to determine whether the observed abnormalities represented persistent deficits or incomplete recovery from more severe initial dysfunction. Future prospective longitudinal cohort studies with standardized serial autonomic assessments at predefined intervals (for example at 1, 3, and 12 months after diagnosis) are needed to better characterize the trajectory, predictors, and long-term clinical significance of dysautonomia in GBS. The inclusion of additional assessments of dysautonomia, such as evaluations of gastrointestinal and urinary autonomic dysfunction, would provide a more comprehensive assessment of autonomic involvement and a better overall clinical overview.

## 5. Conclusions

Autonomic dysfunction may be present in GBS patients beyond the acute phase, even in the absence of overt autonomic symptoms. In our cohort, objective evidence of autonomic impairment was identified in a substantial proportion of patients through comprehensive autonomic testing, highlighting that dysautonomia may remain underrecognized during recovery. Given that autonomic dysfunction, if not identified and managed early, may contribute to significant morbidity and mortality, these findings support the incorporation of objective autonomic function assessment into the follow-up evaluation of patients with GBS.

## Figures and Tables

**Table 1:** Summary of study results: demographics, clinical characteristics and results of autonomic tests.

Variable/Tests	Total
Number of patients	17
Age in years: mean ± SD	58.7 ± 14.1
Mean time since diagnosis to follow-up: mean ± SD	15.5 ± 13.2 months
Sex: Male/female	8/9
Antecedent infection	Respiratory infection 53%
	Gastrointestinal infection 18%
GBS Subtypes	8 (47%) AIDP
	3 (18%) AMAN
	2 (12%) MFS
	3 (18%) GBS/MFS
	1 (5%) Not characterised
Treatment	IVIG 70%
	IVIG/PLEX 24%
	PLEX 6%
ICU admission	2 (13%)
GBS-DS at onset: mean ± SD	3.4 ± 0.8
GBS-DS at discharge: mean ± SD	3.0 ± 1.0
GBS-DS at follow up: mean ± SD	1.1 ± 1.2
At least 1 abnormal autonomic test	7/17 (41%)
Fulfilling criteria for dysautonomia	3/17 (18%)
Heart rate response to standing (30:15 ratio)	4/17 (24%)
Systolic Blood pressure response to standing	3/17 (18%)
Diastolic Blood pressure response to standing	4/17 (24%)
Deep breathing (E/I ratio)	1/17 (6%)
Sustained isometric handgrip	2/17 (12%)
Valsalva manoeuvre	0/14 (0%)
Abnormal Sympathetic skin response (SSR)	2/17 (12%)

Abbreviations: SD, standard deviation; GBS, Guillain–Barré syndrome; AIDP, acute inflammatory demyelinating polyneuropathy; AMAN, acute motor axonal neuropathy; MFS, Miller Fisher syndrome; IVIG, intravenous immunoglobulin; PLEX, plasma exchange; ICU, intensive care unit; GBS-DS, GBS Disability Scale; E/I ratio, expiration-to-inspiration ratio; SSR, sympathetic skin response.

**Table 2:** Demographic characteristics, clinical features, and autonomic nervous system assessment findings for all patients.

P.	Age	Sex	Comorbitities	GBS Subtype	Prodromal Infection	CSF Cells	CSF Protein (mg/dL)	ICU Admission	Treatment	GBS-DS at Onset	GBS-DS at Discharge	GBS-DS at FU	Months Since GBS	Heart Rate Response to Standing	Blood Pressure Response to Standing	E/I Ratio	Sustained Isometric Handgrip	Valsalva	EWING Battery Score *	SSR	Hands Mean ESC	Feet Mean ESC
**1**	46	F	Migraine	Miller Fisher	Yes	0	65	No	IVIG	4	3	0	20	Normal	Normal	Normal	Normal	Normal	0	Normal	63	68
**2**	75	F	HC, HTN	AIDP	Yes	1	65	No	IVIG	4	4	0	20	Normal	Normal	Normal	Normal	Normal	0	Normal	73	68
**3**	40	F	None	Miller Fisher	Yes	1	42	No	IVIG	3	2	0	8	Normal	Abnormal	Normal	Normal	Normal	1	Normal	59	65
**4**	71	M	DM, CAD, HC	AIDP	No	1	103	No	IVIG, PLEX	4	4	3	8	Abnormal	Abnormal	Normal	Abnormal	Aborted	3	Abnormal Hand and Foot	78	58
**5**	38	M	None	AIDP	Yes	1	127	No	IVIG	4	3	0	31	Normal	Normal	Normal	Normal	Normal	0	Normal	87	80
**6**	62	F	HTN, HC	MFS/GBS	Yes	2	142	No	IVIG	4	4	0	22	Abnormal	Normal	Normal	Normal	Normal	1	Normal	86	64
**7**	63	F	None	AIDP	Yes	5	145	No	IVIG	3	3	2	2	Normal	Normal	Normal	Normal	Aborted	0	Normal	77	77
**8**	68	F	CAD, HTN, CKD, Hypothyroidism	MFS/GBS	No	3	36	No	IVIG	2	2	1	20	Normal	Normal	Normal	Normal	Normal	0	Normal	79	81
**9**	66	M	CAD, HTN, HC	AMAN	Yes	1	32	No	IVIG	4	3	0	10	Abnormal	Abnormal	Normal	Normal	Normal	2	Normal	82	50
**10**	36	F	None	AIDP	Yes	2	304	No	IVIG	3	2	0	15	Normal	Normal	Normal	Abnormal	Normal	1	Normal	72	56
**11**	43	M	HTN	AMAN	Yes	4	134	No	IVIG	4	4	1	45	Normal	Normal	Normal	Normal	Normal	0	Normal	81	81
**12**	56	M	HTN, HC	AMAN	Yes	2	57	Yes	IVIG, PLEX	4	4	1	10	Normal	Normal	Normal	Normal	Normal	0	Normal	85	67
**13**	47	M	None	AIDP	Yes	8	222	No	IVIG	2	1	1	3	Normal	Normal	Normal	Normal	Normal	0	Normal	74	64
**14**	64	M	Prostate cancer, CKD	AIDP	No	1	56	No	PLEX	3	2	2	2	Normal	Normal	Normal	Normal	Normal	0	Normal	70	77
**15**	74	F	HC	AIDP	No	5	153	No	IVIG	2	2	2	3	Normal	Normal	Normal	Abnormal	Normal	1	Normal	57	70
**16**	77	F	Osteoporosis	MFS/GBS	Yes	7	221	No	IVIG, PLEX	4	4	4	6	Abnormal	Abnormal	Abnormal	Normal	Aborted	3	Abnormal Foot	78	68
**17**	71	M	None	Unspecified	Yes	2	74	Yes	IVIG, PLEX	4	4	1	40	Normal	Normal	Normal	Normal	Normal	1	Normal	84	73

P, Patient; M, Male; F, female; HC, hypercholesterolemia; HTN, hypertension; DM, diabetes mellitus; CAD, coronary artery disease; CKD, chronic kidney disease; GBS, Guillain–Barré syndrome; AIDP, acute inflammatory demyelinating polyneuropathy; AMAN, acute motor axonal neuropathy; MFS, Miller Fisher syndrome; MFS/GBS, Miller Fisher/Guillain–Barré overlap syndrome; CSF, cerebrospinal fluid; ICU, intensive care unit; IVIG, intravenous immunoglobulin; PLEX, plasma exchange; GBS-DS, GBS Disability Scale; FU, follow-up. 30:15 ratio, heart rate response to standing; E/I ratio, expiration-to-inspiration ratio during deep breathing; SSR, sympathetic skin response; ESC, electrochemical skin conductance. * Definite autonomic dysfunction was defined as ≥2 abnormal Ewing tests.

## Data Availability

The data presented in this study may be made available by the corresponding author upon reasonable request.
